# Supplementary material for: Identification of photoperiod-induced specific miRNAs in the adrenal glands of Sunite sheep (Ovis aries)
Source: Front Vet Sci. 2022 Jul 22;9:888207. doi: 10.3389/fvets.2022.888207 (PMC9354845; doi:10.3389/fvets.2022.888207)
Supplement: Supplementary file 1 [file Data_Sheet_1.ZIP › Supplementary file/miRDeep2 file.zip!/miRDeep2 file/Know_Structure/SP42A1/oar-mir-541.pdf]

miRBase precursor : oar-mir-541
Total read count : 135
oar-miR-541-5p read count : 127
oar-miR-541-3p read count : 8
remaining reads : 0

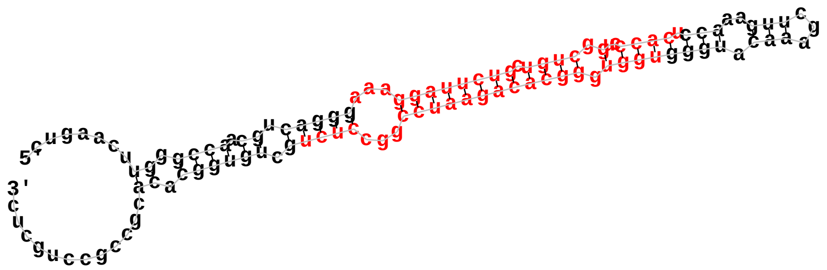

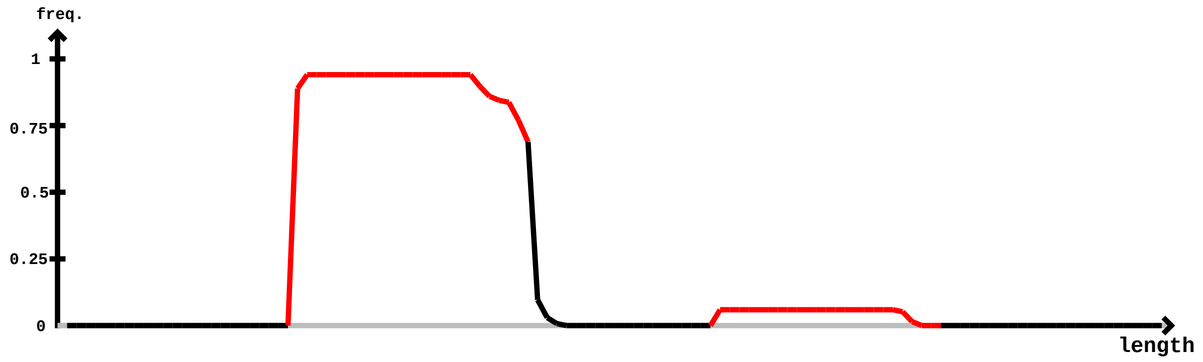

oar-miR-541-3p

oar-miR-541-5p

```
5'- cugaacuugggccaacgucagggaaaggauucugcugucggucccacuccaaaguucgaaacauggguggugggcacagaauccggccucugcuguggcacacgccgccugcuc   -3'    exp
    ........((((..(((.(((((...((((((((.((((.(..((((.(((..(((...))).))))))))).))))))))))))...))))).)))(((....)))))))....    reads       mm         sample
    .......................aaaggauucugcugucggu........................................................................    6           0          seq
    .......................aaaggauucugcugucgguc.......................................................................    5           0          seq
    .......................aaaggauucugcugucggucc......................................................................    2           0          seq
    .......................aaaggauucugcugucgguccc.....................................................................    1           0          seq
    .......................aaaggauucugcugucgguccca....................................................................    9           0          seq
    .......................aaaggauucugcugucggucccac...................................................................    11          0          seq
    .......................aaaggauucugcugucggucccacu..................................................................    76          0          seq
    .......................aaaggauucugcugucggucccacuc.................................................................    6           0          seq
    .......................aaaggauucugcugucggucccacucc................................................................    3           0          seq
    .......................aaaggauucugcugucggucccacucca...............................................................    1           0          seq
    ........................aaggauucugcugucggucccacu..................................................................    4           0          seq
    ........................aaggauucugcugucggucccacuc.................................................................    3           0          seq
    ...................................................................uggugggcacagaauccgg............................    1           0          seq
    ...................................................................uggugggcacagaauccggc...........................    5           0          seq
    ...................................................................uggugggcacagaauccggcc..........................    2           0          seq
```
